# Explaining forest productivity using tree functional traits and phylogenetic information: two sides of the same coin over evolutionary scale?

**DOI:** 10.1002/ece3.1456

**Published:** 2015-03-27

**Authors:** Alain Paquette, Simon Joly, Christian Messier

**Affiliations:** 1 Center for Forest Research, Université du Québec à MontréalP.O. Box 8888, Centre-ville Station, Montréal, QC, H3C 3P8, Canada; 2 Institut de recherche en biologie végétale, Jardin botanique de Montréal and Université de Montréal4101 Sherbrooke East, Montréal, QC, H1X 2B2, Canada; 3 Institut des Sciences de la Forêt tempérée, Université du Québec en Outaouais58 Rue Principale, Ripon, QC, JOV 1V0, Canada

**Keywords:** Biodiversity–ecosystem functioning, conservation, evolutionary distance, functional diversity, phylogenetic diversity, phylogenetic information of species traits

## Abstract

Given evidences that diverse ecosystems provide more services than depauperate ones, much attention has now turned toward finding meaningful and operational diversity indices. We ask two questions: (1) Does phylogenetic diversity contain additional information not explained by functional traits? And (2) What are the strength and nature of the correlation between phylogeny and functional traits according to the evolutionary scale considered? We used data from permanent forest plots of northeastern Canada for which these links have been demonstrated and important functional traits identified. We show that the nature of the relationship between traits and phylogeny varies dramatically among traits, but also according to the evolutionary distance considered. The demonstration that different characters show phylogenetic autocorrelation at different evolutionary depths suggests that phylogenetic content of traits may be too crude to determine whether phylogenies contain relevant information. However, our study provides support for the use of phylogenies to assess ecosystem functioning when key functional traits are unavailable. We also highlight a potentially important contribution of phylogenetics for conservation and the study of the impact of biodiversity loss on ecosystem functioning and the provision of services, given the accumulating evidence that mechanisms promoting diversity effects shift over time to involve different traits.

## Introduction

The importance of biodiversity for ecosystem functioning and for the provisioning of services to humanity is well established (Cardinale et al. 2012; Balvanera et al. 2014). Three components of biodiversity are commonly used to test these links: species richness (SR), functional diversity (FD), and phylogenetic diversity (PD). These components can intuitively be related, and previous studies have indeed shown such relationships (Cadotte et al. 2009, 2013; Paquette and Messier 2011), whereas others did not and argue for complementarity between the different facets of biodiversity (Kluge and Kessler 2011; Cianciaruso et al. 2012; Perronne et al. 2014). If the link between SR and either FD or PD is relatively well understood, the one between FD and PD, and thus the role evolutionary history may play in ecosystem functioning, is much less understood and recently attracted interest (Cadotte 2013; Pavoine et al. 2013; Winter et al. 2013). Functional diversity and PD are expected to be related as functional traits are often characterized by polygenic inheritance, which generally implies that closer species have more similar traits than distant species (Kelly et al. 2014). However, the links are never that simple due in part to the possibility of convergence in traits between distantly related species or divergent selection between closely related species (Coyne and Orr 2004; Cianciaruso et al. 2012; Kelly et al. 2014). The relationship between FD and PD is evolutionary and functionally even more complex and nonlinear because (1) traits do not necessarily evolve at the same rate (e.g., Donoghue 2008), (2) the relationship is likely to be specific to the community and function studied (Cianciaruso et al. 2012; Pavoine et al. 2013); and (3) the number of traits actively involved may well differ somewhat between ecosystem functions. Indeed, disentangling the relationship between FD and PD to understand carbon exchange in prairies might be different from oil degradation in oceans, for example. Thus, the use of PD in biodiversity–ecosystem functioning research (B-EF) and as a conservation tool remains ambiguous due to a lack of a solid conceptual basis and empirical support (Mouquet et al. 2012; Srivastava et al. 2012; Pavoine et al. 2013; Winter et al. 2013) and because it does not guaranty the conservation of the “most diverse set of biological features” (Kelly et al. 2014).

Few high-level integrative mechanisms have been proposed to explain observed B-EF relationships (Loreau and Hector 2001; Loreau et al. 2012). Complementarity effects that promote species coexistence and a more complete use of resources include niche partitioning and facilitation, whereas selection effects relate to dominant species with particular traits (functional identity) (Roscher et al. 2012). Both effects relate to functional traits which link species to the role they play in the ecosystem, shape interactions between plants and their environment, and drive ecosystem processes (Díaz et al. 2004). Much of recent B-EF research now makes use of functional traits and FD to design experiments and analyze responses for its increased power of explanation, but most importantly to get better insight into the possible lower-level biological mechanisms or processes (e.g., light-capture partitioning; Sapijanskas et al. 2014) that lead to the high-level integrative mechanisms (complementarity and selection) (Tobner et al. 2014).

Applications of phylogenetics in ecology and B-EF science, for example through the use of phylogenetic diversity indices (PD) as a proxy for FD, imply the existence of a relationship between the phylogeny and traits (Flynn et al. 2011; Srivastava et al. 2012; Swenson et al. 2012; Pavoine et al. 2013). This implied relationship has made the use of phylogenetic diversity very promising in B-EF science because (1) high-quality phylogenies with complete taxon sampling are easier than ever to obtain (Joly et al. 2014); and (2) good-quality trait data are, in contrast, costly and time-consuming to gather and still scarce and patchy at best, or even nonexistent for some species, ecosystems, or traits (Winter et al. 2013). For example, whereas many have advanced that niche complementarity in plant communities should take place to a good extent through root interactions, few species × root trait data are available (Tobner et al. 2013). Another issue is that the use of functional trait data is often hampered by proprietary claims, as opposed to DNA information that is publicly available. Given the fast rate of democratization of phylogenetic techniques, PD could represent a useful tool for the testing of B-EF hypotheses in many ecosystems for which little trait information is available. This could be particularly important for quantifying the effect of biodiversity loss on many ecosystem services at wide scales over many biomes as shown by the recent implementation of the International Platform on Biodiversity and Ecosystem Services (IPBES) (Balvanera et al. 2014). For these reasons, and because of the relationship of the functional traits of species with their phylogenetic history, PD has been hypothesized to integrate some of the variability in traits among species and used as proxy for FD (Flynn et al. 2011; Cadotte 2013; but see also Perronne et al. 2014 for negative results) and has indeed demonstrated its capacity of seizing a good portion of variance in ecosystem functioning and community assembly (Flynn et al. 2011; Srivastava et al. 2012; Cadotte 2013; Cadotte et al. 2013), although negative results have also been reported (Kluge and Kessler 2011; Cianciaruso et al. 2012; Pavoine et al. 2013). In contrast, good predictive results with FD can only be achieved when good-quality data on relevant traits are available.

Presently, little is known of the relationship between PD and FD in explaining a given ecosystem function (EF) (Flynn et al. 2011; Winter et al. 2013). If both are indeed strong predictors of EF, do they explain the same fraction, or are they complementary? How well can phylogenetic history explain functional trait variation within a community? Although we know that morphological similarity generally decrease with phylogenetic distance (Kelly et al. 2014), we do not know how the similarity of specific traits is correlated with phylogenetic distances in a given ecosystem function context, nor if these patterns are consistent among traits. The studies that investigated the phylogenetic signal contained in traits at a community level found mixed results: whereas there appeared to be little phylogenetic information in traits studied in temperate and tropical tree communities (Swenson et al. 2012), investigation of plant grassland community showed a much stronger influence of phylogeny on traits in one instance (Kembel and Cahill 2011) but not in another (Perronne et al. 2014). But to our knowledge, no study has investigated how functional traits covary with the phylogeny at different phylogenetic distances. Ignoring this aspect affects our understanding of the links between traits and phylogenies because a trait with high rate of evolution would likely contain little phylogenetic information when a large phylogeny is considered, even if it is highly correlated at lower phylogenetic levels. Given the time scale considered in evolutionary history and the number and diversity of evolutionary lineages present in many systems (such as forests, that contain both angiosperms and gymnosperms), it is perhaps not surprising that such links were found to be weak at best in previous studies. Yet, a better understanding of the nature of the relationship between functional traits and phylogenetic relationship is critical in order to provide stronger arguments for the use of PD as a proxy for FD in predicting ecosystem functioning.

Another aspect that has been lacking from previous studies is the proper context within which to interpret the relationships between FD and PD. Indeed, if the comparison is to be relevant for a given community, it is important that the traits studied perform significant functions in that ecosystem. In a previous study linking forest productivity and tree diversity of temperate and boreal forests in Québec, eastern Canada, we found that biodiversity components, including FD and PD, explained a large part (40%) of tree productivity (Paquette and Messier 2011), but we did not parse out these components, nor explore the links between them. This study is aimed at those questions and also benefits from a new, improved and updated molecular phylogeny. The large impact of biodiversity on forest productivity and the identification of the traits that relate significantly with functions results in an ideal system to study the nature of the correlation between FD and PD. The key drivers already identified for forests productivity are wood density (Swenson and Enquist 2007; Chave et al. 2009), “leaf economics” traits (e.g., leaf N content) (Wright et al. 2004; Shipley et al. 2006; Kembel and Cahill 2011), and seed mass (Ben-Hur et al. 2012), a proxy for life-history strategies, together constituting a “plant economics spectrum” that would be defined by a trade-off between fast and slow strategies (Westoby et al. 2002; Reich 2014).

The present study is organized around two central, unanswered questions:

1. Does PD contain additional information from that explained by traits, possibly accounting for unmeasured traits and associated processes?
2. What are the strength and the nature of the correlation between the phylogeny and functional traits of tree and large shrub species that have a significant impact on the functioning of northeastern American forests?

We hypothesize that in the present context where key traits of forest productivity are identified, PD should account for little if no additional variance compared to FD. Also, we expect the relationship between phylogenetic information and functional traits to vary among traits according to the evolutionary scale considered.

## Materials and Methods

### Forest survey data, tree productivity, and environmental controls

We used the permanent forest survey dataset from the province of Québec (eastern Canada) to identify the different biodiversity elements that impact on tree productivity (Paquette and Messier 2011). This sampling effort covers all public lands of the province, from temperate forests in the south to the vast boreal forests of the north, and is thus representative of some of the most extensive terrestrial biomes. Forest biomass growth increments were taken from Paquette and Messier (2011) (see Appendix S1). Briefly, from the complete dataset of over 36,000 plot measurements available, we selected 12,333 pairs of surveys (two contiguous measures of the same plot), for which the plot had not been altered by significant disturbance, and estimated the following:

1. Average total basal area for each species, to be used as abundance and presence/absence table for species, functional and phylogenetic diversity indices, as well as a proxy for competition intensity once all species are summed up (total basal area).
2. Tree productivity, specifically total annual aboveground biomass increments, calculated from biomass estimated using allometric equations.

### Phylogeny reconstruction

We searched in Genbank (www.ncbi.nlm.nih.gov) and BOLD (Ratnasingham and Hebert 2007) for sequences from the plant bar-coding loci (Hollingsworth et al. 2009) for the trees present in the permanent plots of Québec (Appendix S2-2). Details on the sequence alignment, nucleotide substitution model choice, and phylogenetic analyses are provided in Appendix S1. The phylogenetic tree (Appendix S3) was reconstructed in BEAST (Drummond et al. 2012), a useful software for estimating phylogenetic diversity because it reconstructs tree chronograms where branches are proportional to divergence times and where the tips are equidistant from the root. Because no fossil calibrations were used, the divergence times are proportional to their evolutionary or genetic distances, allowing for the testing of our hypotheses.

### Functional and phylogenetic diversity

We assembled a table of functional traits for the 61 tree and large shrub species present in our dataset (Appendix S2-2). We used only continuous traits that had demonstrated links to forest productivity: maximum average height (maxH), wood density (Wd), log seed mass (Sm), and leaf nitrogen content per unit mass (N) (Paquette and Messier 2011; Ruiz-Benito et al. 2014). We chose diversity metrics that were mathematically independent of species richness (SR) to isolate their singular effects. Functional dispersion (FDis) (Laliberté and Legendre 2010) was used to compute a single index of functional diversity (FD) based on maxH, Wd, and Sm and previously identified as the best index for this dataset (Paquette and Messier 2011), to estimate complementarity effects. Leaf N content was included to test for a selection or (functional) identity effects as community weighted mean (CWM—weighted by species basal area), as demonstrated in Ruiz-Benito et al. (2014) (Appendix S1). CWM was considered as an index of diversity, as often done in comparing communities (Diaz et al. 2007). Shade tolerance (an aggregate life-history type of trait) was not used in the calculation of diversity indices, but it was included in the steps where we explore the links between functions and phylogeny.

Phylogenetic diversity (PD) was computed using the phylogenetic species variability index (PSV) (Helmus et al. 2007; Winter et al. 2013), for the same reason of independence from SR (Appendix S1). Plots composed of a single species were assigned FD and PD values of zero. All diversity index computations were carried out within the “R” environment (R Development Core Team 2008) using the “FD” and “picante” libraries (Kembel et al. 2010; Laliberté and Shipley 2011) (see Appendix S1 for further details, and D for R scripts).

### Effect of the different components of diversity on forest growth

Using log-transformed annual aboveground biomass increment of trees as response variable, variance partitioning was used to determine the fractions that could be attributed to the single and combined effects of FD and PD using adjusted *R*^2^ ratios (Peres-Neto et al. 2006). The probabilities associated with the different fractions were computed using partial regressions and 999 permutations of the residuals under the reduced model, except for the common fraction that cannot be tested. These analyses were carried out within “R” using the “vegan” and “venneuler” libraries (Wilkinson 2011; Oksanen et al. 2013).

### Phylogenetic information of functional traits

The phylogenetic content of each trait was estimated using Pagel’s *λ* (1999). To test whether the data contained sufficient information for Pagel’s *λ* estimation, we estimated the 95% confidence intervals (CI) using parametric bootstrapping (1000 simulations) (Boettiger et al. 2012). We then investigated the nature of the autocorrelation of traits at different phylogenetic scales (distances) using Moran’s I autocorrelation coefficient (Moran 1950; Gittleman and Kot 1990). Moran’s I quantifies, in a similar fashion as the Pearson correlation, the autocorrelation between species values of a trait. To investigate how the autocorrelation varies at different phylogenetic distances, we used an approach analogous to spatial autocorrelograms by evaluating Moran’ I autocorrelation statistics at eight different phylogenetic distance classes (Legendre and Legendre 1998). Practically, this means that the autocorrelation at a specific distance class only considers species pairs that diverged within a given distance range. A positive Moran’s I value at a given class indicates that species of that class tend to have more similar traits than when all species are considered, whereas a negative value suggest the opposite. Comparative analyses were performed in R using the “ape” (Paradis 2005), “picante”, “geiger” (Harmon et al. 2008), “pmc” (Boettiger et al. 2012), “phylobase” (Hackathon et al. 2014), and “adephylo” (Jombart et al. 2010) libraries. Analyses were performed on all 61 tree species present in the Québec surveys including 12 less common species that were not present in the plots retained for growth analysis (Appendix S2-2). Excluding those less common species did not change the outcome of the analyses (not shown).

## Results

### Growth and diversity

Approximately 72% of the total variance in forest productivity was explained when all environmental factors as well as biodiversity indices were considered in a multiple regression model where each variable contributed significantly (*P* < 0.01) to the total (Table A1) (adapted from Paquette and Messier 2011). FD (FDis) and PD (PSV) complementarity indices were thus still significant after accounting for the biotic and abiotic environment (total basal area, depth of the organic horizons and mean annual temperatures) and community functional identity (CWM of N content of leaves). Biodiversity was found to explain a large proportion of forest productivity with complementarity (FD and PD) and identity (CWM) components together explaining 47%. Functional diversity (FDis) was the best model predictor of forest growth among biodiversity components of complementarity. Phylogenetic species variability (PSV) explained a lesser proportion of forest productivity than the other indices (22%) but was still significant (Table A1). Henceforth, we used PSV and FDis to evaluate the importance of PD and FD, respectively, because they are computationally unaffected by species richness and thus facilitate the interpretation of the results (see Table A3 for a detailed analysis of correlations between diversity indices).

**Figure 1:**
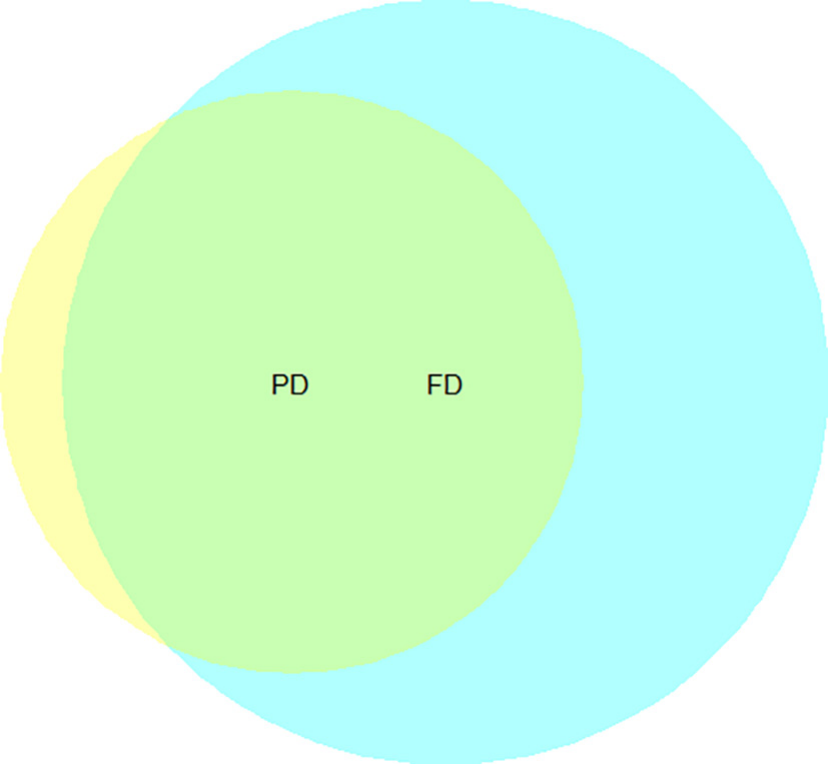
Venn diagram of variation partitioning between complementarity components of functional (blue) and phylogenetic (yellow) diversity. The amount of variance explained (adjusted *R*^2^ fractions) by each predictor is represented by the relative size of the circles (0.38 and 0.22, respectively; labels mark centers), the overlapping area is variance that is jointly explained by both predictors (0.20; green), while nonoverlapping areas indicate variance that is uniquely explained by a single predictor (0.18 and 0.02, respectively). The total amount of variance explained was therefore 0.40, and residuals were 0.60. All testable fractions highly significant (*P* = 0.001; *N* = 12,333). See Appendix S1 for further details and all fractions (Table A4).

Variance partitioning between functional and phylogenetic diversity revealed that both components together explained roughly 40% of the total variance (Fig.1). Half of that (20%) was shared between the two, but the singular FD fraction accounted for the better part of the remaining variance explained (18%), leaving little to the singular PD contribution (2%). In all, only about half of the variability in functional traits that explained forest growth could be captured by variance in evolutionary distance.

### Phylogenetic content of traits

**Figure 2:**
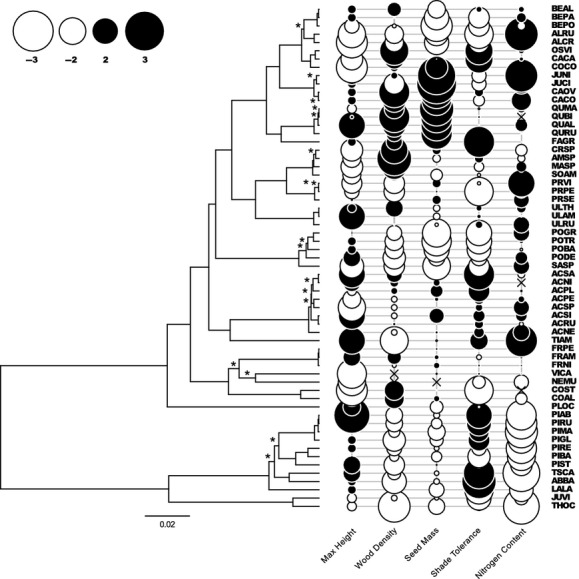
Standardized values (bubble size and shade) of the four functional traits and shade tolerance along with the Bayesian phylogeny of the 61 tree and large shrub species with known presence in the Québec dataset. All branches have posterior probability of 1.0, except for those marked by an asterisk (see Appendix S3 for the full tree including branch length and probability). See Appendix S2-2 for species’ acronyms.

A simple graphical representation of trait values into the phylogeny of species clearly illustrates that species functional traits are linked to the evolutionary history of species (Fig.2). Functionally similar species tend to be closely related, as is the case, for example, for the small seeded, r-selected *Betulaceae* and the large seeded, K-selected *Fagaceae* and *Juglandaceae*. Even though shade tolerance was not included in B-EF analyses because it is not a functional trait per se but rather the result of several traits (i.e., an aggregated trait), it was plotted beside the phylogeny as it is a key ecological characteristic of species often used in explaining forest dynamics. Shade tolerance also illustrates parallel adaptation with both angiosperms and gymnosperms containing shade-tolerant and shade-intolerant species. Figure2 also reveals that whereas traits do vary with the phylogeny, they do not appear to do so at the same level, with some traits being conserved over much larger taxonomic groups (e.g., N content) than others (e.g., maximum height).

To estimate the phylogenetic signal in functional traits, we used Pagel’s *λ*. Whereas maximum height had a very low *λ*, which suggests independence from the phylogeny, all other traits were found to have relatively important phylogenetic content (Table1). However, the wide 95% CI obtained by parametric bootstrapping suggests that it is difficult to confirm the presence of phylogenetic signal in seed mass and N content even though their *λ* is relatively large.

**Table 1:** Phylogenetic information in functional traits using Pagel’s *λ* (1999; ±95% confidence intervals)

Trait	Pagel’s *λ* (95% CI)
Max height (maxH)	0.0176 (1×10^−7^; 0.0393)
Wood density (Wd)	0.7929 (0.3616; 0.8965)
Seed mass (Sm)	0.2281 (1×10^−7^; 0.3629)
Shade tolerance	0.8230 (0.4097; 0.8780)
Leaf N content (N)	0.6503 (1.166×10^−7^; 0.8224)

**Figure 3:**
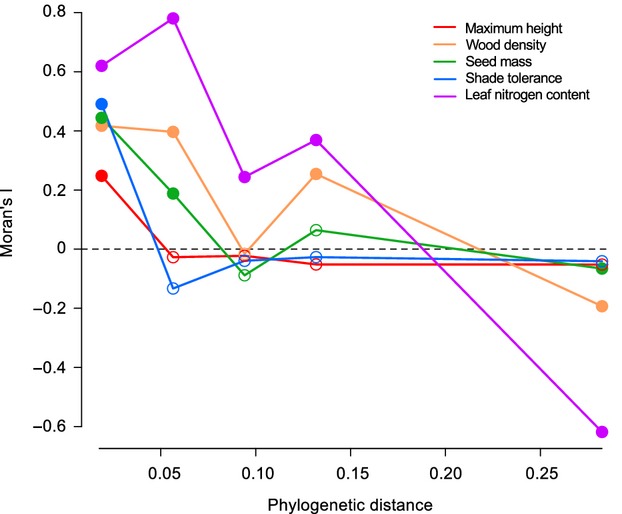
Moran’s I phylogenetic correlogram for the four functional traits plus shade tolerance for eight phylogenetic distance classes for 61 tree species. Distance classes with small values consist of species of recent divergence. Filled circles indicate significant correlations. There were no comparisons possible for three classes because of large differences between Divisions.

To further investigate the nature of the phylogenetic signal in functional traits, we used a Moran’s I autocorrelogram (Fig.3). All traits had a significant Moran’s I statistic at the smallest phylogenetic distance, suggesting that trait values tend to be similar for closely related species (tip of the phylogeny). But at larger phylogenetic distances, traits showed different patterns. For instance, N content and wood density showed significant negative correlations at the largest distance class, suggesting a strong difference between gymnosperms and angiosperms for these traits. Moreover, these two traits showed positive autocorrelations at intermediate distance classes, suggesting that species within angiosperms and gymnosperms tend to have similar values. In contrast, shade tolerance showed a significant correlation only at the smallest distance. Finally, maximum height showed a significant negative autocorrelation at the second distance class, which suggests a rapid evolutionary turnover for this character as closely related species are negatively correlated. The Moran’s I phylogenetic autocorrelogram was also performed with different number of classes, but the results always showed the same trends (not shown).

## Discussion

This and other studies concur that forest tree productivity is strongly and positively affected by biodiversity (e.g., Vilà et al. 2013; Ruiz-Benito et al. 2014), making forests an ideal system to further study the relationships between FD and PD. We first hypothesized that in situations where key traits determining ecosystem functioning are known and available (such as for forest productivity), PD would account for little additional variance explained compared to FD alone. Recently, Cadotte et al. (2013) showed that using both components enhanced the capacity to detect assembly patterns in alpine plant communities, arguing for complementary information. Our results concur, but further show that the proportion that could be attributed singly to PD was small, confirming our hypothesis. The relative contributions of PD and FD was also investigated by Cadotte et al. (2013) but using a different approach; they included a parameter assigning weight to the trait versus phylogenetic components and showed different patterns for the assemblage of contrasted alpine communities. Given the hypothesis that trait information should be included at least partially in the phylogeny and that PD might explain additional variance only when trait information is incomplete, we argue that the variance partitioning approach is an interesting alternative because it explicitly accounts for (1) the common fraction explained by both components; and (2) the fractions that could be attributed singly to either traits, or evolutionary processes alone (and thus possibly to unmeasured traits).

Our second hypothesis, that the relationship between phylogenetic information and functional traits should vary among traits according to the evolutionary scale considered, was also confirmed (Fig.3). The present context was ideal to investigate the nature of those links because most of the information provided by PD is also contained in FD. As expected and found elsewhere (Kelly et al. 2014), trait values tended to be similar for closely related species. However, we also found that different traits did not vary similarly along an evolutionary distance gradient. For instance, whereas wood density and leaf N content were found to be similar across large taxonomic groups, it was the opposite for max height and shade tolerance. For these latter traits, only closely related species shared similar values and the phylogenetic signal was lost at greater divergence. This is important as previous studies did not account for scale in exploring the relation between functional traits and the phylogeny of species, which may in part explain their relatively pessimistic opinions regarding the use of phylogenies in B-EF research. Indeed, Pagel’s *λ* was often not significant when the whole phylogeny was considered (Table1). This suggests that this statistic, applied over the whole tree, might be too crude to judge whether some of the information contained in a trait is also present in phylogenetic relationships. Consequently, one should be careful in interpreting genetic signal of trait when considering a large phylogeny, as an apparent lack of signal for the whole phylogeny may hide a strong signal at lower divergence, as was observed here for maximum height and seed mass.

Our results also help define where and why phylogenetics could be used as a proxy for FD as underlined by Winter et al. (2013). Indeed, a phylogeny could be seen as a mixture of the effects of different traits evolving at different speeds. As at least three functional traits were shown to be important for forest productivity, and those traits were also shown to vary at different depth in evolutionary history, the phylogeny can be seen as a composite that accounts for that variability in both trait space and evolutionary distance and be used as proxy for functional diversity.

In a context where the relevant traits are relatively well known, such as with the present study, it is expected that functional diversity will explain a greater proportion of the total variance than phylogenetic diversity (as well as better relate to processes—see below). But phylogenetic information may still be used advantageously in cases where data on traits are scarce or incomplete to accelerate the investigation of B-EF relationships in undocumented ecosystems, or at wide continental scales. The exact relationships are probably context specific, but a relationship between PD and FD should exist wherever the functional traits of importance have a genetic basis. Also, as more studies report how each species’ contributions to B-EF become increasingly singular with time (producing an increasingly linear relationship with SR and reducing functional redundancy), following the insurance hypothesis, evolutionary distance approaches may become even more relevant and powerful, being more inclusive of each species’ past, present and future contributions (Allan et al. 2011; Reich et al. 2012). Although specific relationships between FD and PD are likely context specific, the approach presented in this study is general and we have no reason to believe that extending the use of PD as a proxy for FD to approximate the sensitivity of ecosystem functions to diversity losses would produce biased results, albeit incomplete.

We also note that whereas almost all of phylogenetic diversity effect on ecosystem function could be captured by functional diversity, the opposite was not true. The fact that functional diversity contained some unique information is important and points out that good quality and most importantly relevant functional traits are still stronger predictors of EF than is phylogenetic information alone, as noted elsewhere (Pavoine et al. 2013). Traits are also critical if one wishes to identify low-level mechanisms (e.g., light-capture partitioning) that lead to complementarity or identity effects. For temperate and boreal forests, the hypothesis that documented traits might be incomplete and that missing traits would be included in PD was thus not supported. This does not mean, however, that no other trait plays an active role in processes leading to the complementary use of resources. Only controlled experiments will be able to isolate and test the hypothesized processes at play (Tobner et al. 2014). Here, we could only assess that the traits used (relating to aboveground structure and life-history strategy) do relate to complementarity in forest biomass production. If other processes (e.g., belowground) that involved other traits also contributed to complementarity, they were captured by the former set of traits. For example, late-successional tree species, which in our dataset would be those with larger seeds and denser wood, also tend to have larger root:shoot ratios and deeper root systems. Another example is that late-successional species would discriminate N-forms in favor of the less costly ammonium (as opposed to nitrate) due to their lesser overall N demand and energy-limited, low-light environment (Fredeen and Field 1992; Templer and Dawson 2004).

There is clearly renewed interest for phylogenetic comparative methods as ecologists delve into the past to better understand how it shaped the present state of ecosystems, such as community composition or selection on traits in communities (Davies et al. 2012; Peres-Neto et al. 2012; Cadotte et al. 2013). More studies are required to further understand the relationship between phylogenetic history and functional traits in more systems, as noted by Winter et al. (2013) and Pavoine et al. (2013). Despite these limitations, the rapid increase in availability of molecular data makes it possible for ecologists to estimate B-EF effects, their direction, strength and shape, in any ecosystem for which molecular data are available, provided that the traits expected to drive the function being investigated do have genetic content. This is important as we lack functional traits for many organisms of less studied ecosystems, functions or services, and even more so for novel or urban ecosystems (Nock et al. 2013). Another justification for the use of phylogenetic information in B-EF research is the recent demonstration of the fading of functional redundancy between species over time (Allan et al. 2011; Reich et al. 2012). If further confirmed, this may reveal that mechanisms promoting complementarity may shift over time to involve different and often unknown or difficult to assess traits, promoting niche partitioning at different levels. This is a fundamental issue for conservation and restoration efforts, as proposed recently by Cadotte (2013) based on results from experimental plant assemblages, but it also highlights a potentially important contribution of phylogenetics to the long-term study of the impact of biodiversity loss on ecosystem functioning and the provision of services. Although evolutionary history may not help in identifying the specific mechanisms at play at any given time, it does include, at least in part, all possible mechanisms as it covers the natural history of trait evolution.

## Supporting Information

**Appendix S1.** Further details regarding the dataset and methods used, and additional results. **Appendix S2.** Tables containing (1) functional trait metadata, (2) list of species in the dataset and their functional trait values, and (3) GenBank references. **Appendix S3.** Nexus phylogeny, including matK and rbcL sequences. **Appendix S4.** R scripts used to compute functional and phylogenetic diversity indices, test the relationships between traits and the phylogeny, and perform variance partitioning analyses.
