# Supplementary material for: Explaining forest productivity using tree functional traits and phylogenetic information: two sides of the same coin over evolutionary scale?
Source: Ecol Evol. 2015 Mar 27;5(9):1774–83. doi: 10.1002/ece3.1456 (PMC4485959; doi:10.1002/ece3.1456)

Appendix A: Further details regarding the dataset and methods used, and additional results.

*Description of the dataset*

The original study details can be found in Paquette and Messier (2011); details relevant to the present study are recalled here. We used the permanent plots of the Québec (eastern Canada) forest survey dataset, dating back to 1970 and still in service today (Duchesne and Ouimet 2008). The survey data are available from the *Géoboutique* at *Québec's Ministère des Ressources naturelles* (http://geoboutique.mrn.gouv.qc.ca).

This forest sampling effort covers all of the Province public lands, thus including some of the most extensive ecosystems on Earth, from temperate forests to the vast boreal forests of the north (Fig. A1). New permanent plots are added every year while older ones are re-measured approximately every ten years (mean 9.96 years, standard deviation 3.21 years for our subset). From that large dataset of over 36,000 plot measurements we selected pairs of surveys (two contiguous measures of the same plot) that met our criteria, namely:

- The plot had not been affected by a significant natural disturbance between the two censuses considered. “Significant disturbance” was defined as having killed >25% of trees (based on basal area).

- The plot had not been subjected to human interventions of any kind, including plantations.

A number of environmental variables are also collected at the same time (see MRNFQ 2006). Only continuous type environmental descriptors (log-transformed when necessary) that could be evaluated consistently in the field and showed significant correlations with forest productivity

were kept for analyses, namely: drainage class, slope, and the pH and depth of the LFH (leaf, litter, humus) organic horizons. To control for climate we used plot coordinates to compute elevation and climatic variables using ANUSPLIN interpolation of 30-year normals from all available weather stations in Québec and adjacent territories (Hutchinson 1995, Milewska et al. 2005). Since latitude, longitude and elevation were used in the computation of climate variables, they were excluded from further analyses.

For all 12,324 pairs of censuses we then computed:

1) Average total basal area for each of the species present. This was used as a matrix of species abundances and presence/absence for species, functional, and phylogenetic diversity indices, as well as a proxy for competition intensity once all species are summed-up (total basal area).

2) Forest productivity, specifically annual aboveground biomass increments, computed using tree DBH and the following equation:

$$Y = \frac{\sum_{i=1}^{n} b_{i-t2} - b_{i-t1}}{t2 - t1} \tag{A.1}$$

Where $Y$ is total aboveground biomass yearly increment of live trees between a pair of measurements $t1$ and $t2$ for a given plot, $b$ is the biomass of tree $i$ present at both sampling times. Individual biomasses $b$ of trees were computed using DBH and published equations for aboveground stem and branch biomass (Lambert et al. 2005). For the few species without published equations, as well as for the very few trees identified as “unknown” in the surveys, we

either used the “general” (all species, i.e. for “unknowns”), the “conifer” or the “hardwood” equations provided therein.

Trees that died between sampling times, as well as recruits, were not included since we could not determine when a tree died or when it passed the 9.1cm threshold, and is further supported by Lei *et al.* (2009) not finding a significant relationship between diversity and mortality or recruitment in spruce forests of neighboring New Brunswick (eastern Canada). Therefore *Y* is the annual biomass increment of live trees present at both sampling times for a given pair of plot measurements.

*Functional diversity computation details*

We used the same FD and PD indices as in the previous paper (Paquette and Messier 2011), from which important and relevant to the present study details are reported here. We used a newly developed R package (R Development Core Team 2008) to compute indices of functional diversity from the table of traits (Appendix B). The “FD” package (Laliberté and Legendre 2010) computes a Gower dissimilarity matrix (Gower 1971) and can handle species relative abundances, trait weights, missing data, and all types of continuous, ordinal and binary data, including asymmetric binary. The procedure handles ordinal variables as recommended by Podani (1999).  A new index called functional dispersion (FDis) (Laliberté and Legendre 2010) was used because of the many characteristics that made it appropriate for our dataset. FDis uses multivariate dispersion (Anderson et al. 2006) as a multidimensional index of functional dispersion and is thus the average distance of individual species to the centroid of all species in the functional trait space, weighted by their relative abundances if available. It has a number of desirable properties as an FD index, among which independence from species richness (SR). It is

conceptually similar and correlated with Rao's quadratic entropy (Botta-Dukát 2005, Laliberté and Legendre 2010). Like Rao’s Q, but unlike many other indices such as functional divergence (Mason et al. 2005, Villéger et al. 2008), FDis can compute values for communities with two or more singular species (species that differ in at least one trait). That was an important feature for us working with temperate and boreal forests where monocultures and two-species communities are common. Plots composed of a single species were assigned an FD (and PD) value of zero. All diversity index computations were carried out within the 'R' environment (R Development Core Team 2008) using the 'FD' and 'picante' libraries (Kembel et al. 2010, Laliberté and Shipley 2011). R scripts are available in Appendix D.

For the present study we used the FD index that performed best at explaining tree productivity based on our previous result, built using three traits: maximum height of trees, wood density, and seed mass (Appendix B). We also included species richness for reference. Since this time we were interested in the relationships between traits and the phylogeny, we also added two more traits to the above three. Although shade tolerance, an aggregate life-history type of trait, was not used in the calculation of diversity indices to explain productivity, it was included in the following steps where we explore the links between functions and phylogeny. The same was done for leaf N content, for which a link to selection or functional identity effects was demonstrated when used as community weighted mean (CWM) in Ruiz-Benito et al. (2014) as well as in the present study (see below and Table A1). Leaf mass per area was not used because of the difficulty in using a common and comparable methodology for both conifer and broadleaf species, and because it is highly correlated with N content, which is included (Wright et al. 2004).

*Tree reconstruction and phylogenetic diversity computation details*

In contrast with functional traits and functional diversity, which were the same as for the previous study, the present was much more focussed on the phylogeny and phylogenetic diversity (PD), so the molecular phylogeny of tree species growing in Québec was completely rebuilt from the ground up, augmented and updated with new genetic information. We searched again in Genbank (www.ncbi.nlm.nih.gov/) for sequences from the plant barcoding loci, rbcL and matK (Hollingsworth et al. 2009) for the trees present in the permanent plots of Québec (Appendix B). When a sequence was not available for the exact same species for either marker, we used sequences available for species of the same genus, when possible of species from the same section and natives to North America (details in Appendix B). Four other species were included to cut long branches in the phylogeny and obtain a topology congruent with that of the APGIII (Bremer et al. 2009). Sequences were aligned with Muscle (Edgar 2004) and did not require further manual editing. Alignments were trimmed to maximise the information while minimizing the number of missing data. Final alignment lengths were of 741 and 1370 base pairs for *matK* and *rbcL*, respectively. The *rbc*L and *mat*K markers were selected because they are the most commonly sequenced for the species of interest and because they provide complementary phylogenetic resolution: *rbc*L evolves more slowly and resolves deeper phylogenetic relationships whereas *mat*K provides better resolution among more closely related species.

The best substitution model for both markers was found to be TVM + G according to the Aikake Information Criterion in jModelTest 0.1.1 (Posada 2008). The phylogenetic analysis was performed in BEAST (Drummond et al. 2012), a useful software for estimating phylogenetic diversity because it reconstructs tree chronograms where branches are proportional to divergence times and where the tips are equidistant from the root. Trees can thus be directly used for

phylogenetic diversity estimation without further modifications. A relaxed uncorrelated lognormal clock (Drummond et al. 2006) was used for both markers. The rate of *matK* (prior gamma(2,1)) was estimated relative to that of *rbcL*. A birth-and-death model was used for the species tree prior; priors for the growth and death rates were both set to a gamma (2,100).

The substitutions model and the molecular clock were unlinked, whereas the tree topology was linked between markers. The Markov Chain Monte Carlo simulation was run for $6 \times 10^6$ generations and the chain was sampled every $2{,}000^{th}$ generations. Chain convergence was confirmed by ensuring that all parameters had converged to the same value in two independent runs, as determined with Tracer (Rambaut and Drummond 2009). Trees from the independent runs sampled after a burn-in of $1 \times 10^6$ were combined to build the maximum clade credibility phylogeny.

We used Bayes Factors estimated according to Suchard et al. (2001) in Tracer to test simpler models for the phylogenetic analysis: strict molecular clock (instead of a relaxed uncorrelated lognormal clock), linked (instead of unlinked) substitution models between markers, and a linked (instead of unlinked) relaxed lognormal clock between markers. None of these simplification resulted in a decisive better model (interpretation follows Kass and Raftery 1995) (Table A2). Final phylogeny used in the present study is provided as Appendix C.

Phylogenetic diversity (PD) indices could then be computed using this phylogeny. Faith (1992) index (fPD) was computed as well as the phylogenetic species variability index (PSV) which quantifies how phylogenetic relatedness decreases the variance of a hypothetical unselected or neutral trait shared by all species in a community (Helmus et al. 2007). PSV is statistically independent of SR and can be considered pure phylogenetic signal that is not confounded with

SR. Both PD indices were computed using the “picante” package for R (R Development Core Team 2008, Kembel et al. 2009). The same tree was also used to test for phylogenetic content in functional traits (detailed in main text). Comparative analyses were performed in R using the 'ape' (Paradis 2005), 'picante', 'geiger' (Harmon et al. 2008), 'pmc' (Boettiger et al. 2012), 'phylobase' (Hackathon et al. 2014), and 'adephylo' (Jombart and Dray 2008) libraries. R scripts for the above are available in Appendix D.

*Further details on analyses performed*

The first step of analyses was to screen for significant explanatory variables (Table A1). This was adapted from our previous study (Paquette and Messier 2011) by the addition of a functional identity index (CWM based on leaf N content). Using log-transformed annual aboveground biomass increment of trees as response variable, we used stepwise regressions to identify the most promising explanatory variables. This was carried out in logical independent steps to identify abiotic and biotic drivers of productivity (Diaz et al. 2007): 1) Abiotic factors (local environmental and climatic conditions; 2) Stand basal area, as proxy for competition intensity; 3) Biodiversity indices related to complementarity (FD and PD); and 4) Biodiversity indices of identity (CWM and SR). Note that Faith's PD and species richness were only included for reference. Because of the many variables available and possible loss of interpretable information with insignificant increases in variance explained, we used Mallow’s Cp to help retain only those variables that contributed the most. Indeed more environmental and climate variables came out significant but added very little to the model and so were dropped from further analyses. Stepwise procedures as well as standard analyses, data handling and transformations were carried out in JMP 7 (SAS Institute Inc. 2007 Cary, NC).

There were significant correlations between the different components of biodiversity (Table A3). PD indices were highly correlated with each other (0.89), the remaining variance being mostly attributable to SR, with which fPD was much more correlated (0.82) than was PSV (0.54), as expected from their computational characteristics. FD did not escape that trend however; even FDis which is mathematically free of SR was still much correlated with it (0.71). Lastly, FD showed correlations with both fPD (0.68) and PSV (0.56). Indeed, outside of controlled experiment it is not possible to distinguish such naturally closely related variables (Tobner et al. 2014).

Finally, we include in Table A4 more details from the variance partitioning method applied to single out the effects of functional and phylogenetic components of diversity. Constrained (those appearing on Fig. 1) as well as unconstrained fractions are shown. These analyses were carried out within 'R' using the 'vegan' package (Oksanen et al. 2013). R scripts available in Appendix D.

## LITERATURE CITED


Anderson, M. J., K. E. Ellingsen, and B. H. McArdle. 2006. Multivariate dispersion as a measure of beta diversity. Ecology Letters **9**:683-693.

Anderson, M. J. and P. Legendre. 1999. An empirical comparison of permutation methods for tests of partial regression coefficients in a linear model. Journal of Statistical Computation and Simulation **62**:271-303.

Boettiger, C., G. Coop, and P. Ralph. 2012. Is your phylogeny informative? Measuring the power of comparative methods. Evolution **66**:2240-2251.

Botta-Dukát, Z. 2005. Rao's quadratic entropy as a measure of functional diversity based on multiple traits Journal of Vegetation Science **16**:533-540.

Bremer, B., K. Bremer, M. Chase, M. Fay, J. Reveal, D. Soltis, P. Soltis, and P. Stevens. 2009. An update of the Angiosperm Phylogeny Group classification for the orders and families of flowering plants: APG III. Botanical Journal of the Linnean Society **161**:105–121.

Diaz, S., S. Lavorel, F. de Bello, F. Quetier, K. Grigulis, and T. M. Robson. 2007. Incorporating plant functional diversity effects in ecosystem service assessments. Proceedings of the National Academy of Sciences **104**:20684–20689.

Drummond, A. J., S. Y. W. Ho, M. J. Phillips, and A. Rambaut. 2006. Relaxed phylogenetics and dating with confidence. PLoS Biology **4**:e88.

Drummond, A. J., M. A. Suchard, D. Xie, and A. Rambaut. 2012. Bayesian phylogenetics with BEAUti and the BEAST 1.7. Molecular Biology and Evolution **29**:1969-1973.

Duchesne, L. and R. Ouimet. 2008. Population dynamics of tree species in southern Quebec, Canada: 1970-2005. Forest Ecology and Management **255**:3001-3012.

Edgar, R. C. 2004. MUSCLE: multiple sequence alignment with high accuracy and high throughput. Nucleic Acids Research **32**:1792-1797.

Faith, D. P. 1992. Conservation evaluation and phylogenetic diversity. Biological Conservation **61**:1-10.

Freckleton, R. P., P. H. Harvey, and M. Pagel. 2002. Phylogenetic Analysis and Comparative Data: A Test and Review of Evidence. The American Naturalist **160**:712-726.

Gower, J. C. 1971. A general coefficient of similarity and some of its properties. Biometrics **27**:857-871.

Hackathon, R., B. Bolker, M. Butler, P. Cowan, D. d. Vienne, D. Eddelbuettel, M. Holder, T. Jombart, S. Kembel, F. Michonneau, D. Orme, B. O'Meara, E. Paradis, J. Regetz, and D. Zwickl. 2014. phylobase: Base package for phylogenetic structures and comparative data. R package version 0.6.3.

Harmon, L. J., J. T. Weir, C. D. Brock, R. E. Glor, and W. Challenger. 2008. GEIGER: investigating evolutionary radiations. Bioinformatics **24**:129-131.

Helmus, M. R., T. J. Bland, C. K. Williams, and A. R. Ives. 2007. Phylogenetic Measures of Biodiversity. The American Naturalist **169**:E68–E83.

Hollingsworth, P. M., L. L. Forrest, J. L. Spouge, M. Hajibabaei, S. Ratnasingham, M. Van Der Bank, M. W. Chase, R. S. Cowan, D. L. Erickson, and A. J. Fazekas. 2009. A DNA barcode for land plants. Proceedings of the National Academy of Sciences **106**:12794-12797.

Hutchinson, M. F. 1995. Interpolating mean rainfall using thin plate smoothing splines. International Journal of Geographical Information Science **9**:385-403.

Jombart, T. and S. Dray. 2008. adephylo: exploratory analyses for the phylogenetic comparative method.

Kass, R. E. and A. E. Raftery. 1995. Bayes factors. Journal of the American Statistical Association **90**:773-795.

Kembel, S., D. Ackerly, S. Blomberg, P. Cowan, M. Helmus, H. Morlon, and C. Webb. 2009. picante: R tools for integrating phylogenies and ecology. R package version 0.6-0.

Kembel, S. W. and J. F. Cahill, Jr. 2011. Independent Evolution of Leaf and Root Traits within and among Temperate Grassland Plant Communities. PLoS ONE **6**:e19992.

Kembel, S. W., P. D. Cowan, M. R. Helmus, W. K. Cornwell, H. Morlon, D. D. Ackerly, S. P. Blomberg, and C. O. Webb. 2010. Picante: R tools for integrating phylogenies and ecology. Bioinformatics **26**:1463-1464.

Laliberté, E. and P. Legendre. 2010. A distance-based framework for measuring functional diversity from multiple traits. Ecology **91**:299-305.

Laliberté, E. and B. Shipley. 2011. FD: measuring functional diversity from multiple traits, and other tools for functional ecology. R package version 1.0-11.

Lambert, M. C., C. H. Ung, and F. Raulier. 2005. Canadian national tree aboveground biomass equations. Canadian Journal of Forest Research **35**:1996-2018.

Lei, X., W. Wang, and C. Peng. 2009. Relationships between stand growth and structural diversity in spruce-dominated forests in New Brunswick, Canada. Canadian Journal of Forest Research **39**:1835-1847.

Mason, N. W. H., D. Mouillot, W. G. Lee, and J. B. Wilson. 2005. Functional richness, functional evenness and functional divergence: the primary components of functional diversity. Oikos **111**:112-118.

Milewska, E. J., R. F. Hopkinson, and A. Niitsoo. 2005. Evaluation of geo-referenced grids of 1961-1990 Canadian temperature and precipitation normals. Atmosphere-Ocean **43**:49-75.

MRNFQ. 2006. Normes d'inventaire forestier: placettes-échantillons permanentes. 2006 edition. Direction des inventaires forestiers, Ministère des Ressources naturelles et de la Faune, Québec, Canada.

Oksanen, J., F. G. Blanchet, R. Kindt, P. Legendre, P. R. Minchin, R. B. O'Hara, G. L. Simpson, P. Solymos, M. H. H. Stevens, and H. Wagner. 2013. vegan: Community Ecology Package. R package version 1.8-2.

Paquette, A. and C. Messier. 2011. The effect of biodiversity on tree productivity: from temperate to boreal forests. Global Ecology & Biogeography **20**:170-180.

Paradis, E. 2005. Statistical analysis of diversification with species traits. Evolution **59**:1-12.

Podani, J. 1999. Extending Gower's General Coefficient of Similarity to Ordinal Characters. Taxon **48**:331-340.

Posada, D. 2008. jModelTest: phylogenetic model averaging. Molecular Biology and Evolution **25**:1253-1256.

R Development Core Team. 2008. R: a language and environment for statistical computing. R Foundation for Statistical Computing, Vienna, Austria.

Rambaut, A. and A. J. Drummond. 2009. Tracer 1.5.

Ruiz-Benito, P., L. Gómez-Aparicio, A. Paquette, C. Messier, J. Kattge, and M. A. Zavala. 2014. Diversity increases carbon storage and tree productivity in Spanish forests. Global Ecology & Biogeography **in press**.

Suchard, M. A., R. E. Weiss, and J. S. Sinsheimer. 2001. Bayesian Selection of Continuous-Time Markov Chain Evolutionary Models. Molecular Biology and Evolution **18**:1001-1013.

Tobner, C. M., A. Paquette, P. B. Reich, D. Gravel, and C. Messier. 2014. Advancing biodiversity – ecosystem functioning science with the use of high-density tree-based experiments. Oecologia **174**:609-621.

Villéger, S., N. W. H. Mason, and D. Mouillot. 2008. New multidimensional functional diversity indices for a multifaceted framework in functional ecology. Ecology **89**:2290-2301.

Wright, I. J., P. B. Reich, M. Westoby, D. D. Ackerly, Z. Baruch, F. Bongers, J. Cavender-Bares, T. Chapin, J. H. C. Cornelissen, M. Diemer, J. Flexas, E. Garnier, P. K. Groom, J. Gulias, K. Hikosaka, B. B. Lamont, T. Lee, W. Lee, C. Lusk, J. J. Midgley, M.-L. Navas, Ü. Niinemets, J. Oleksyn, N. Osada, H. Poorter, P. Poot, L. Prior, V. I. Pyankov, C. Roumet, S. C. Thomas, M. G. Tjoelker, E. J. Veneklaas, and R. Villar. 2004. The worldwide leaf economics spectrum. Nature **428**:821-827.

Table A1: Results of a regression analysis for the most important explanatory variables from each group of environmental (abiotic and biotic) and diversity-derived drivers of tree productivity (log annual growth). Adjusted $R^2$ are given for each variable taken alone as well as globally for each group (underlined).

| Variable | Stepwise (adj$R^2$) |
|---|---|
| Environment | 0.68 |
| Mean temperature | 0.44 |
| Stand basal area (log) | 0.43 |
| Organic layer depth | 0.16 |
| Biodiversity - Complementarity | 0.40 |
| Functional Diversity (FDis) | 0.38 |
| Phylogenetic Species Variability (PSV) | 0.22 |
| Biodiversity - Identity | 0.40 |
| Leaf mass per area (CWM.N) | 0.40 |

Notes. Adapted from Paquette and Messier (2011) by the addition of a CWM index. All results shown were highly significant individually as well as within a global multiple regression model ($p<0.01$; N = 12 333). Species richness (SR) and Faith's Phylogenetic Diversity (fPD) are not shown for clarity and were not included in multiple regressions (and thus group $R^2$) so as not to mask other diversity indices (see above); their adj$R^2$ values were 0.32 for both.

Table A2. Likelihood of the standard model and Bayes factor results of alternative models compared to the best model (the standard). Models are ordered from the best to the worst.

| model | ln P(model\|data) | S.E. | $\log_{10}$ Bayes Factors |
|---|---|---|---|
| Standard | -18358.048 | +/- 0.447 | 0 |
| Linked clock models | -18470.326 | +/- 0.53 | -48.762 |
| Strict molecular clocks | -18509.017 | +/- 0.523 | -65.565 |
| Linked substitution models | -18645.206 | +/- 0.711 | -124.711 |

Following Kass and Raftery (1995), an absolute $\log_{10}$ Bayes Factor > 2 is considered as a decisive statement in favour of the best model.

Table A3. Pearson pairwise correlation coefficients between biodiversity components (all correlations highly significant; N = 12 333 forest plots). SR: species richness; FDis: functional dispersion index for functional diversity; fPD: Faith's phylogenetic diversity index; PSV: phylogenetic species variability index.

| | SR | FDis | fPD | PSV |
|---|---|---|---|---|
| SR | 1 | 0.71 | 0.82 | 0.54 |
| FDis | | 1 | 0.68 | 0.56 |
| fPD | | | 1 | 0.89 |
| PSV | | | | 1 |

Table A4. Variation partitioning between complementarity components of functional (FDis) and phylogenetic diversity (PSV) (adjusted $R^2$). See Figure 1 for the corresponding Venn diagram describing the partitioning of the variation.

| Partition | |
|---|---|
| [a+b] = FD | 0.38 |
| [b+c] = PD | 0.22 |
| [a+b+c] = all (total biodiversity) | 0.40 |
| [a] = FD \| PD | 0.18 |
| [b] (interaction) * | 0.20 |
| [c] = PD \| FD | 0.02 |
| [d] (Residuals) * | 0.60 |

Notes. All testable fractions highly significant (p=0.001; N = 12 333). Fractions marked with * are not testable (Anderson and Legendre 1999). Last four "single" fractions are constrained. For example [a] is the variance explained by FD alone with control for PD.

Figure A1: Map of plots used in this study and their distribution across all bioclimatic domains of continuous forest in Québec, Canada. Originally published in Paquette and Messier (2011).

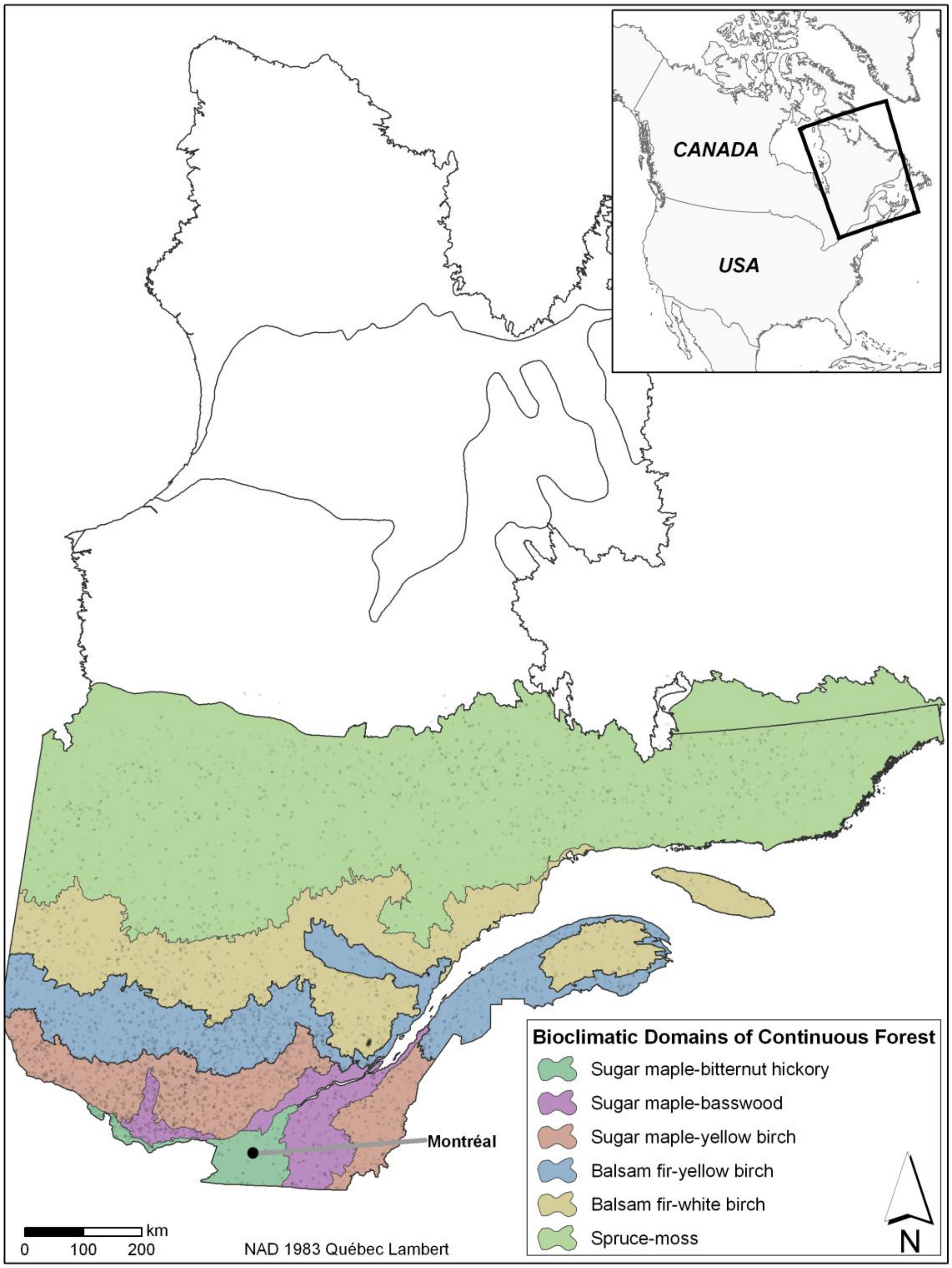

Supplement: Supplementary file 1 [file ece30005-1774-sd1.zip › AppendixA.pdf]
